# Outcomes of Concurrent Hiatus Hernia Repair with Different Bariatric Surgery Procedures: a Systematic Review and Meta-analysis

**DOI:** 10.1007/s11695-023-06914-7

**Published:** 2023-11-02

**Authors:** Henry Mills, Yousef Alhindi, Iskandar Idris, Waleed Al-Khyatt

**Affiliations:** 1 Medical School University of Nottingham, Nottingham, UK; 2 Clinical, Metabolic and Molecular Physiology Research Group, MRC-Versus Arthritis Centre for Musculoskeletal Ageing Research, University of Nottingham, Royal Derby Hospital Centre, Uttoxeter Road, Derby, DE22 3NE UK; 3 National Institute for Health Research (NIHR) Nottingham Biomedical Research Centre, Nottingham, UK; 4 Division of Applied Medical Sciences, University of Hail, Hail, Saudi Arabia; 5 East Midlands Bariatric & Metabolic Institute, Royal Derby Hospital, Derby, DE22 3NE UK; 6 Bariatric & Metabolic Surgery Department of Excellence, Health Point Hospital, A Mubadala Health Partner, Zayed Sports City, United Arab Emirates

**Keywords:** Hiatus hernia repair, Bariatric surgery, Mortality, Re-operation, Gastro-oesophageal reflux disease, GORD

## Abstract

**Background:**

Hiatus hernia (HH) is prevalent among patients with obesity. Concurrent repair is often performed during metabolic and bariatric surgery (MBS), but a consensus on the safety and effectiveness of concurrent HH repair (HHR) and MBS remains unclear. We performed a systematic review of the safety and effectiveness of concurrent HHR and MBS through the measurement of multiple postoperative outcomes.

**Method:**

Seventeen studies relating to concurrent MBS and HHR were identified. MBS procedures included laparoscopic sleeve gastrectomy (LSG), Roux-en-Y gastric bypass (LRYGB), and adjustable gastric banding (LAGB). Studies with pre- and postoperative measurements and outcomes were extracted.

**Results:**

For LSG, 9 of 11 studies concluded concurrent procedures to be safe and effective with no increase in mortality. Reoperation and readmission rates however were increased with HHR, whilst GORD rates were seen to improve, therefore providing a solution to the predominant issue with LSG. For LRYGB, in all 5 studies, concurrent procedures were concluded to be safe and effective, with no increase in mortality, length of stay, readmission and reoperation rates. Higher complication rates were observed compared to LSG with HHR. Among LAGB studies, all 4 studies were concluded to be safe and effective with no adverse outcomes on mortality and length of stay. GORD rates were seen to decrease, and reoperation rates from pouch dilatation and gastric prolapse were observed to significantly decrease.

**Conclusion:**

Concurrent HHR with MBS appears to be safe and effective. Assessment of MBS warrants the consideration of concurrent HHR depending on specific patient case and the surgeon’s preference.

**Graphical Abstract:**

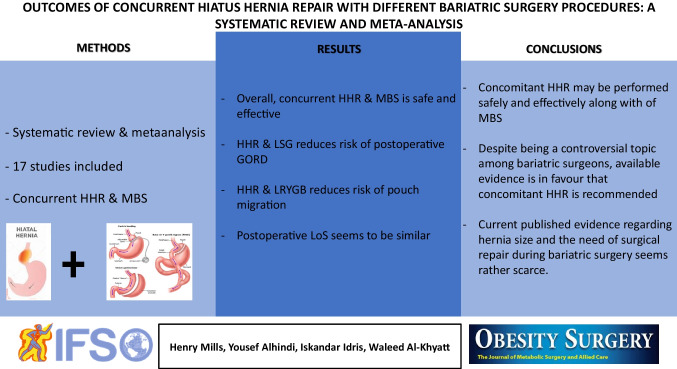

**Supplementary Information:**

The online version contains supplementary material available at 10.1007/s11695-023-06914-7.

## Introduction

Hiatus hernias (HH) are classified as either sliding (type I) hiatal hernia which represents 85–90% of all diagnosed hernias or paraesophageal (types II–IV) hiatal hernias, and they constitute 10–15% of diaphragmatic hiatus hernias. Obesity, defined as BMI of >30kg/m^2^, is associated with a fourfold increased risk of developing HH compared to individuals without obesity [1–3]. In fact, HH are prevalent up to 40% of individuals with obesity [1]. This is largely due to greater intra-abdominal pressure (IAP) 2–3 times that of patients without obesity [2, 3].

Metabolic and bariatric surgery (MBS) is increasingly being utilised in clinical practice and is the most effective strategy to produce and sustain significant weight loss in patients with obesity. Due to the higher prevalence of HH in patients with obesity, HH repair (HHR) is often performed concurrently with MBS. Since MBS results in a significant reduction in IAP, if performed simultaneously with HHR, a reduced recurrence rate should be expected [1, 2]. A study utilising the Metabolic and Bariatric Surgery Accreditation and Quality Improvement Program (MBSAQIP) database reported an HH repair rate of 21.0% in sleeve gastrectomy (LSG) and 10.8% in Roux-en-Y gastric bypass (LRYGB) [4]. Current guidelines recommend concomitant HHR during MBS when the defect is intraoperatively found [5]. Also, the safety profile of a concurrent HHR was suggested to be similar to that of MBS alone [6, 7].

Offering a concurrent HH repair is still a controversial topic among metabolic and bariatric surgeons. In 2022, a survey of the membership of the International Federation of the Study of Obesity (IFSO) reported that only 23% of bariatric surgeons suggested that LSG should not be performed if GORD is present [8]. The National Bariatric Surgery Registry (NSBR), 2020, showed GORD rates increased from 23 to 31% post LSG [9]. However, a systematic review and meta-analysis by Chen *et al.* demonstrated additional HHR to improve GORD resolution and GORD-HRQL in LSG [10]*.* Moreover, it has been observed the rate of concurrent repairs performed with LSG has risen, whereas the rate of concurrent repair being performed with LRYGB remained constant [11].

Laparoscopic Roux-en-Y gastric bypass (RYGB), another widely performed MBS, has been shown to improve GORD symptoms in HH and reduces the need for medical therapy [12, 13]. However, since weight loss per se has also been shown to dramatically reduce IAP, it has been suggested that the use of LRYGB alone could potentially resolve symptoms of GORD without HHR and that performing the additional repair was not required and may result in additional complications [14]. However, a study by Kothari *et al.* reported that combined HHR and LRYGB is associated with no increase in the 30-day mortality and morbidity when compared to LRYGB alone [15]. Furthermore, a study reported improvement in GORD-Related Quality of Life (GORD-HRQL) scores and in the use of anti-reflux medications when combining LRYGB with HHR [16].

Complications however can arise from leaving a HH unrepaired during MBS. In a study using multi-section CT, a migration rate of 37% associated with symptoms of GORD was observed at 1–10 months follow-up [17]. Since the diameter of the sleeve is similar to that of a HH, a migration is observed, leading to anatomical disruption of the lower oesophageal sphincter. Caceres *et al.* have also outlined the risk of roux-limb herniation, post LRYGB, if HH are left unrepaired, leading to gastric pouch incarceration or small bowel obstruction with associated significant morbidity [18].

Whilst concurrent MBS and HHR is therefore recommended to improve clinical outcomes, the safety and effectiveness of concomitant MBS and HHR remain unclear mainly due to issues relating to collating and synthesising current evidence. This systematic review aims to investigate the safety and clinical outcomes of HHR when performed concurrently with different types of MBS to provide an evidence-based guide for surgeons.

## Methods

The Preferred Reporting Items for Systematic Reviews and Meta-Analysis (PRISMA) were applied for this systematic review. This allowed an efficient and reliable recovery of information to achieve the objectives and aims of this study.

### Search Strategy

A literature search was performed on PubMed, Embase, Medline and 2 grey literature databases, OpenGrey and EthOS, on the 10th of October 2022. The search strategy was directed at including studies of concurrent HHR and MBS, with complications and outcomes reported.

The PubMed tool, Mesh, was utilised to broaden the search. The final search for PubMed was the following: (((“Hernia, Hiatal”[Mesh]) OR (hiatus hernia repair)) OR (Paraesophageal Hiatal Hernia)) AND ((“Bariatric Surgery”[Mesh]) OR metabolic surgery OR weight loss surgery). The full search is shown in Supplement 1. The final search using Ovid for Medline and Embase is shown in Supplements 2 and 3. There were no restrictions for geographic location, ethnicity, or gender.

### Inclusion and Exclusion Criteria

The studies included had to meet the following criteria: (1) observational studies (retro/ prospective studies), (2) full-text publication, (3) adult patients, (4) English Language, (5) articles reporting outcomes of concomitant HHR and MBS, (6) first-time HHR, and (7) first-time MBS. Comments, systematic reviews, no abstract, animal studies, cross-sectional studies, case studies, case reports and studies with <10 patients were all excluded.

Duplicates were removed via Endnote. Two authors (YA & HM) began study selection on Rayyan through the examination of the title and the abstract. Any conflict was discussed and resolved. Studies which were selected then underwent data extraction and quality assessment.

### Data Extraction

Two authors (YA & HM) independently reviewed the title, abstract, and full-text publication on the inclusion and exclusion criteria. The following data was extracted using a pre-constructed data extraction form: intervention, author, year, country, type of study, number of patients, age, measured outcomes, and follow-up. Outcome measures which were extracted through the same process were pre- and postoperative BMI, excess weight loss (EWL), pre- and postoperative GORD, reoperation rate, 30-day readmission, blood loss, mortality, infection and length of stay (LoS). This data was recorded using Microsoft Excel.

### Quality Assessment

The Joanna Briggs Institution (JBI) quality assessment tool for critical appraisal was applied by the researcher to evaluate the studies included. The JBI tool provided clear explanations and questions to test the reliability, validity and relevance of the corresponding cohort studies. The researcher used the guidance provided by the JBI tool explanation to address the chance of bias in the studies’ design and judgement (Supplement 4).

A high-quality study was to include a comparator and have a follow-up of at least 30 days and use appropriate statistical analysis. At least 30 days were required to ensure the recording of postoperative outcomes, including 30-day reoperation and readmission.

### Meta-analysis

A meta-analysis of results was performed on outcome measures where data was consistent. Review Manager 5.4.1 was used for this meta-analytical investigation. Odds ratios (OR) were formed using the statistical method of Mantel-Haenszel and a fixed effect analysis model, where 95% confidence intervals were created to determine the range of values where the true mean lies. Forest plots were then exported to the review.

## Results

### Literature Search Results, Study Characteristics, and Quality Assessment

**Fig. 1:**
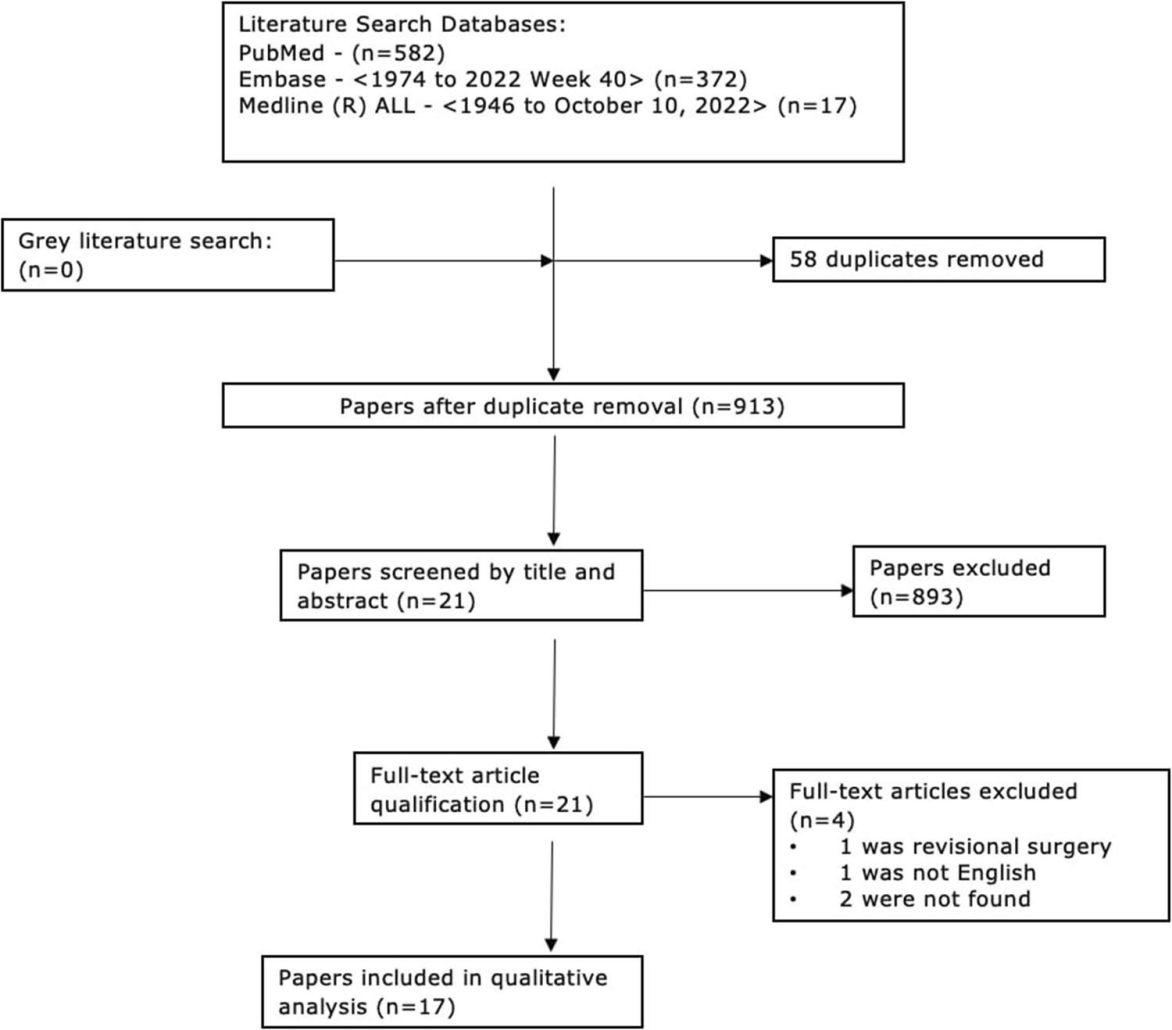
Flow diagram of the study selection procedures

Nine hundred seventy-one studies were obtained from the final database searches. After removal of duplicates, 913 studies were left. After screening for relevant articles, 21 studies were left, these underwent full paper screening, and 17 final papers were eligible and included in the final systematic review. The flow diagram of the study selection procedures is summarised in Fig. 1. Two studies that were not found were not full publication but rather a conference proceedings/abstract.

Of the 17 articles included, six were prospective observational studies and eleven were retrospective. Six studies compared the impact of MBS and HHR on GORD. The number of participants per study ranged from 10 to 50,951. The characteristics of included studies are summarized in Table 1.

**Table 1:** Study characteristics

Studies	Country	Surgical technique	Design	Number (% Female)	Age (years)	Pre-op BMI (kg/m^2^)	Follow-up	PreGORD	PostGORD	GORD remission	Reoperation	Readmisison	Bleeding	Mortality	Infection	Length of stay	Quality assessment
Shada [11]	USA	LSG	Retrospective	4021 (84.82%)	47 (39–56)	43.1 (39.6–47.6)	30 days				19/1812	58/ 1895	50	4	67	1.9 days ± 1.2	9
Shada [11]	USA	LRYGB	Retrospective	1935 (84.82%)	47 (39–56)	43.1 (39.6–47.6)	30 days				44/1469	111/1753	50	4	67	1.9 days ± 1.2	9
Kothari [15]	USA	LRYGB	Retrospective	644 (NA)	NA	NA	30 days					1.55%		0		2.97 ± 5.29 days	5
Samakar [19]	USA	LSG - Posterior repair using nonabsorbable sutures	Prospective	58 (74.1%)	49.5 ± 11.2	44.2 ± 6.6	97.5 weeks (44-172)	26/58	22/58	4				0/58			5
Soricelli [20]	Italy	LSG - Posterior repair	Prospective	97 (87.2%)	NA	44 ± 3.5	18 months	41/97	8/97	33							7
Santonicola [21]	Italy	LSG - Posterior repair	Prospective	78 (87.2%)	39.3 ± 1.4	44.6 ± 0.7	NA	30/78	24/78	6							6
Aridi [22]	Lebanon	LSG	Retrospective	4687 (84.3%)	46.1 ± 10.8	44.6 ± 6.8	30 days	47/76	49/76	-2	56		28	5	15	1.8 days ± 1.5	6
Garg [23]	India	LSG - Posterior repair, nonabsorbable sutures	Retrospective	10 (50%)	42.6 ± 14.39	45.83 ± 9.28	11.70 ± 6.07 months	5/10	1/10	4				0			5
Boru [24]	Italy	LSG - Posterior repair	Prospective	48 (NA)	NA	NA	59.1 ± 9.1 months	17/48	6/38	11	6 conversion LRYGB				3		8
Boru [24]	Italy	LSG - Bioabsorbable Mesh, Posterior repair	Prospective	48 (NA)	NA	NA	60.2 ± 7.8 months	20/48	9/46	11	2 conversion LRYGB						8
Janik [25]	USA	LSG	Retrospective	50,951 (83.45%)	NA	44.17 ± 6.64	30 days	34.19			1.10%	4.01%	0.63%	0.02%	0.35%		9
El Chaar [26]	USA	LSG - Anterior Cruroplasty (Type I)	Retrospective	56 (78.5%)	48.1 ± 10.2	42.6 ± 5.9	6 months				0	1		0			7
El Chaar [26]	USA	LSG - Posterior Cruroplasty, with or without synthetic absorbable mesh (Type II-IV)	Retrospective	43 (86%)	49.2 ± 9.1	43.5 ± 5.7	6 months				1	1		0			7
Lewis [27]	USA	LSG	Retrospective	1546 (83.3%)	43.3 ± 10.3	NA	1-3 years (74.1% - 43.4%)	83.40%			4.20%	0.80%					11
Lewis [27]	USA	LRYGB	Retrospective	457 (87.5%)	44.2 ± 10.5	NA	1-3 years (70.1% - 35.9%)				6.40%						11
Pham [28]	USA	LSG — posterior repair, biologic mesh used for patients with attenuated tissues	Prospective	23 (95.7%)	53.4 (37–66)	41.9	6.16 months (1–19)					2	0	0	0	2.83 days	7
Al-Haddad [29]	USA	LRYGB — anterior or posterior	Retrospective	1945 (88%)	47.42 (46.1–48.8)	NA	NA	55%						0			7
Al-Haddad [29]	USA	LAGB — anterior or posterior	Retrospective	1959 (82%)	49.2 (47.8–50.5)	NA	NA	44%						0			7
Al-Khyatt [30]	UK	LRYGB — posterior crural suture repair	Prospective	24 (NA)	54 (48–59)	52 ± 8.1	12 months						1				7
Ardestani [31]	USA	LAGB	Retrospective	8120 (83.2%)	47.6 ± 11.9	44.3 ± 6.3	10 months (0-39)	49% (3979)	26.46% (2111)	1868	0.40%			0.02%		0.7 days	7
Long [32]	Australia	LAGB — anterior or posterior, or a combination	Prospective	114 (87.7%)	51.8 (26.4–69.8)	41.3 (30.5–64.2)	4.9 years (0.7-10.5)	56/111	34/69	22	4						7
Gulkarov [33]	USA	LAGB — posterior repair, non-absorbable sutures	Retrospective	520 (74.8%)	46 ± 11.7	44.6 ± 7.16	20.5 months				18/520 (3.46%)					24.5 ± 6.8 h	7

The quality of the cohort studies varied between studies. The average score was 7.06/11 using the JBI quality assessment tool; the quality assessment score is displayed in Table 1.

### Meta-analysis of Outcomes

#### Effect of Concurrent Laparoscopic Sleeve Gastrectomy (LSG) + HHR

##### GORD

**Fig. 2:**
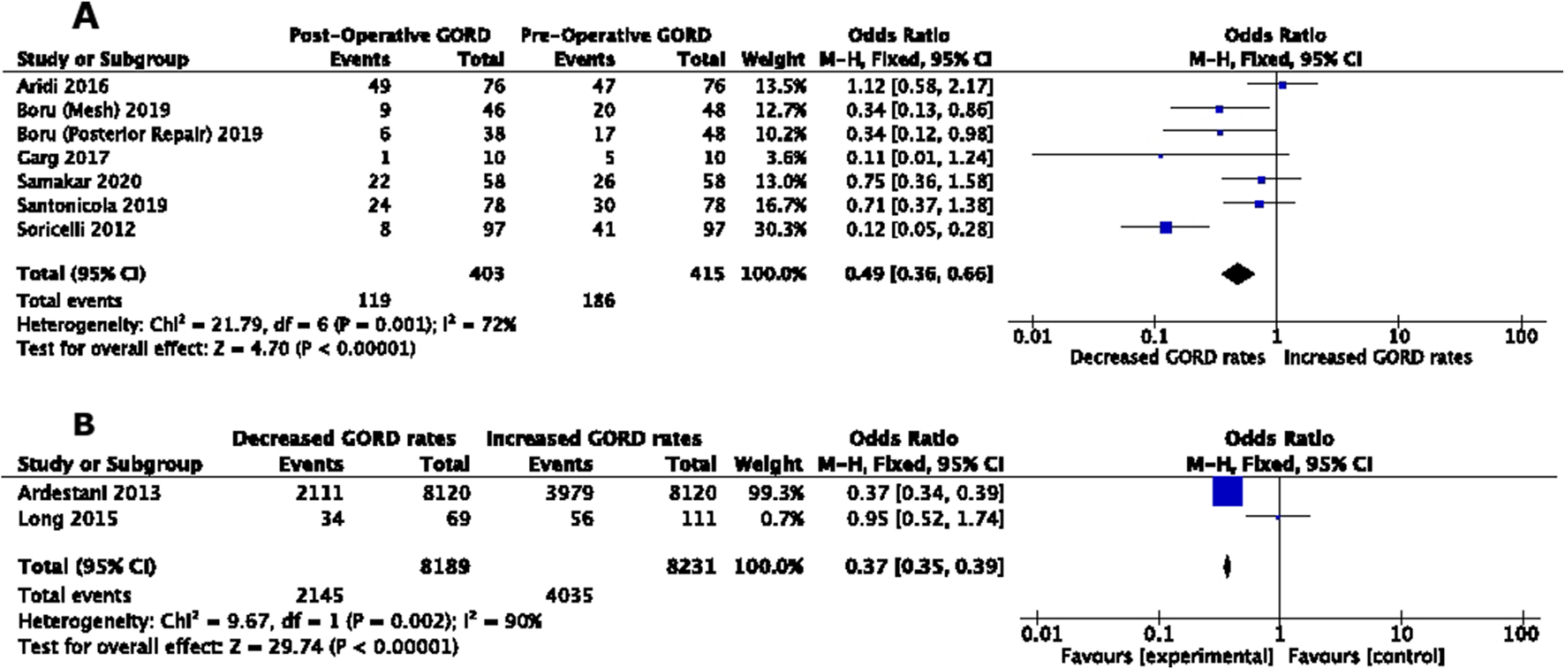
Meta-analysis of outcomes related to GORD following concurrent hiatus hernia repair with LSG (**A**) and LAGB (**B**) procedures

Six trials involving a total of 415 patients reported the incidence of GORD rates after LSG+HHR. The incidence of GORD remission ranged from an increase of 4% to a decrease of 80%. A fixed-effect model was used to pool the results; the heterogeneity between the studies was significant (*I*^2^ = 72%, *P* = 0.001). The results of this meta-analysis showed that the incidence of GORD remission was 51% (OR = 0.49; 95% CI, 0.36, 0.66; *P*=0.0001) (Figure 2A) [19–24].

##### Infection

Compared to the control group, infection rates was reported to increase within the concurrent HHR group in Shada *et al.* [11] and Janik *et al.* [25], but it was noted to decrease among concurrent HHR group in Dakour Aridi *et al.* [22]; however, the results did not largely differ.

##### Bleeding

Bleeding was reported in comparison with a control in two studies: Dakour Aridi *et al.* [22] and [25]. Dakour Aridi *et al.* [22] showed less risk of bleeding within the concurrent HHR group, whilst Janik *et al.* [25] showed no difference in bleeding rates in either group.

##### Reoperation

**Fig. 3:**
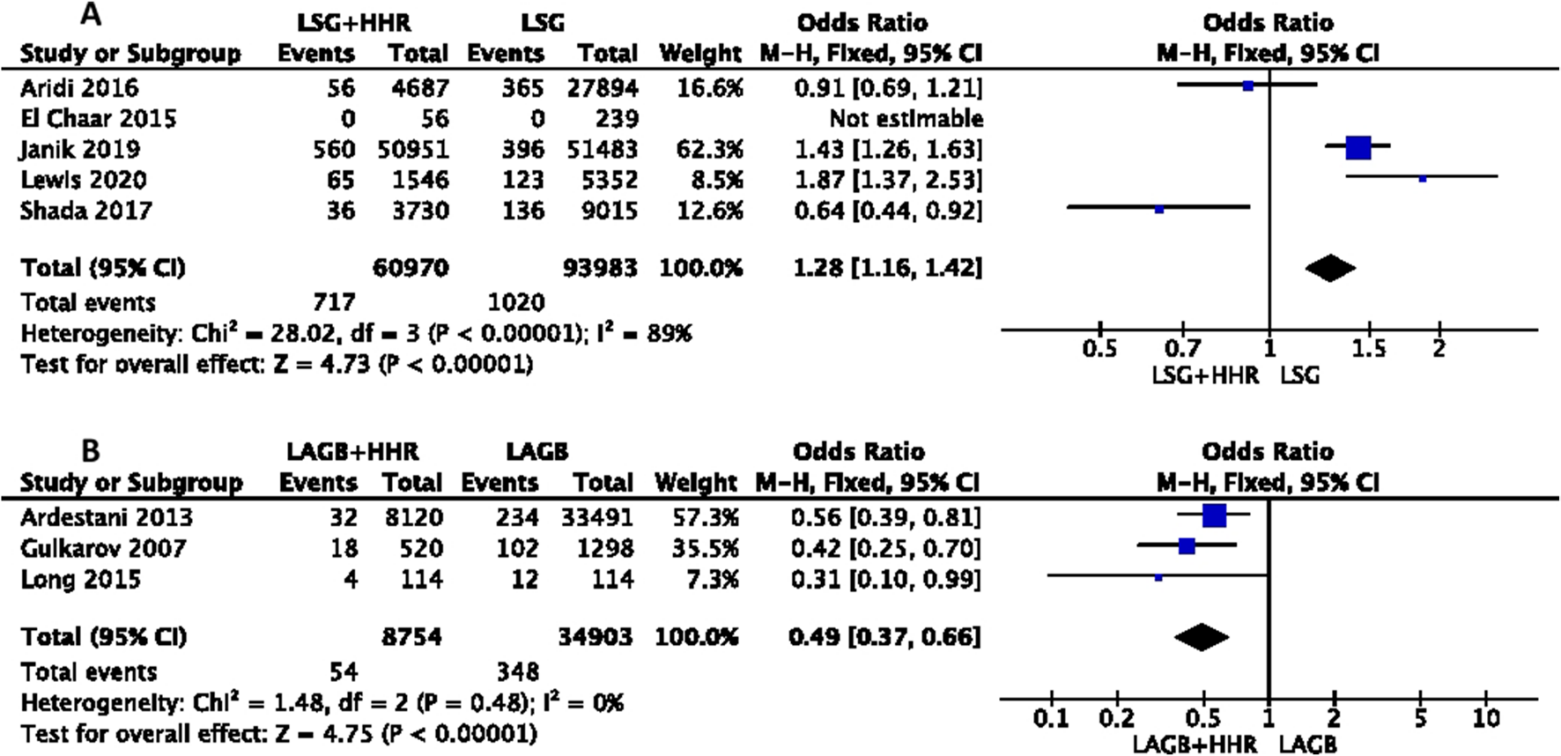
Meta-analysi of outcomes related to re-operation following concurrent hiatus hernia repair with LSG (**A**) and LAGB (**B**) procedures

Five trials involving 93983 patients reported the rate of reoperation in LSG+HHR and a control LSG alone group. The rate of reoperation ranged between 36 and 83% in the LSG+HHR group. A fixed-effect model was used to pool the results. There was significant heterogeneity between the studies (*I*^2^ = 89%, *P* ≤ 0.00001). This meta-analysis showed the pooled reoperation rate was 28% greater with concurrent HHR (OR = 1.28; 95% CI, 1.16, 1.42) (Figure 3A) [11, 22, 25–27].

##### Mortality

**Fig. 4:**
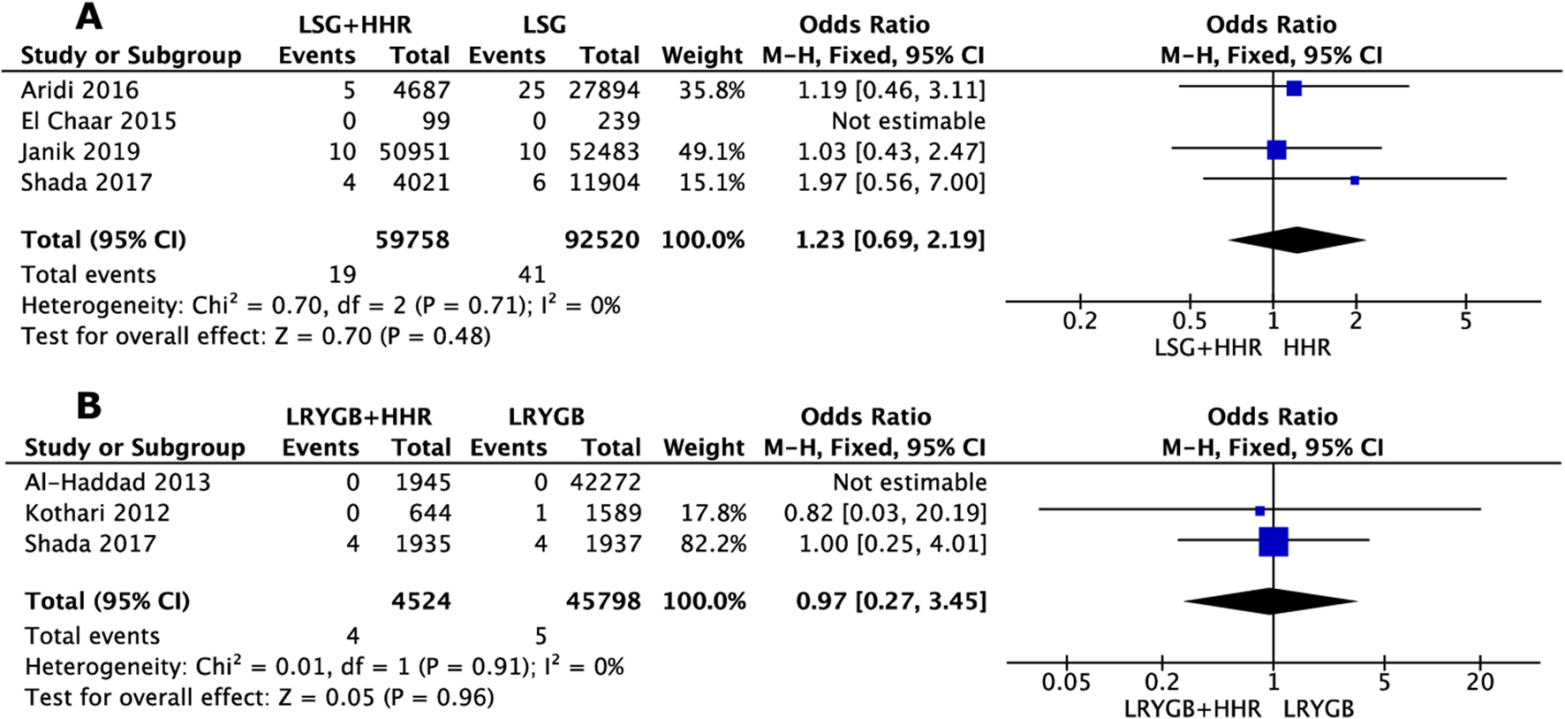
Meta-analysis of outcomes realted to mortality following concurrent hiatu hernia repair with LSG (**A**) and LRGYB (**B**) procedures

Four trials involving 92520 patients reported the rate of mortality in LSG+HHR and a control LSG alone group. The heterogeneity between the studies was not significant (*I*^2^ = 0%, *P* = 0.71). The overall effect of concurrent HHR increased mortality rates by 23% (OR = 1.23; 95% CI, 0.69, 2.19) (Figure 4A) [11, 22, 25, 26].

##### Readmission

In this review, 3 trials involving 62322 patients compared the rate of readmission between LSG+HHR and a the LSG alone group. The heterogeneity between studies was significant (*I*^2^ = 89%; *P* < 0.00001). The overall effect of concurrent HHR increased readmission rates by 30% (OR = 1.30; 95% CI, 1.22, 1.39) (Supplement 5).

##### Length of Stay

Length of stay (days) was reported in comparison with a control in Protyniak *et al.* [28] and Shada *et al.* [11], of which duration was not significantly affected by concurrent HHR.

##### Safety and Feasibility

Of the 11 studies, 9 demonstrated that t concurrent HHR and LSG is both safe and feasible without any significant increase in complication rates. Samaker *et al.* demonstrated an increase in GORD rates so did not support concurrent HHR with LSG [19]. Santonicola *et al.* saw no improvement in outcomes, so thought the additional HHR to be unnecessary [21].

#### The Effect of Concurrent Roux-en-Y-Gastric Bypass (LRYGB) + HHR

##### GORD

As expected, there were no observed changes in GORD rates for concurrent HHR + LRYGB group compared to LRYGB alone group.

##### Mortality

Three trials including 45798 patients reported the rate of mortality in both LRYGB+HHR and LRYGB alone groups. The heterogeneity between studies was not significant (*I*^2^ = 0%; *P* < 0.91). The overall effect of concurrent HHR demonstrated a decrease mortality rates by 3% (OR = 0.97; 95% CI, 0.27, 3.47) compared to LRYGB group (Figure 4B) [11, 15, 29].

##### Reoperation and Readmission

Reoperation was only recorded in only 2 trials [11, 27]. Lewis *et al.* demonstrated the risk of several complications to be more than twice as likely in LRYGB than in LSG (short-term morbidity: 6.20% versus 2.69%) (reoperation: 3.00% versus 1.05%) (readmission: 6.33% versus 3.06%). However, this was stated to be likely due to the higher rates of morbidity in patients undergoing LRYGB compared to LSG as previously identified in studies without HHR. This study found similar morbidity and mortality for the concurrent procedure when compared to MBS alone. Reoperation was observed to increase in [27] with HHR compared to LSG alone control [27].

Shada *et al.* found overall morbidity and readmission rates were significantly greater for the among undergoing MBS alone. In their subgroup analysis, there was significant improved overall morbidity and lower readmissions and reoperations in the HH repair + LSG patients compared to HH repair + LRYGB patients [11].

##### Bleeding

Bleeding was recorded in only 2 trials where there was no significant difference in bleeding rate was recorded in LRYGB with or without concurrent HHR [11, 30].

##### Infection

Infection was recorded in only 1 trial. However, the results were that of the combined number in LGS+HHR with LRYGB+HHR; therefore, no outcomes could be concluded.

##### Length of Stay

Length of stay was recorded with a comparison in 1 trial. Study [28] observed no significant difference with concurrent HHR when performed with LRYGB.

#### The Effect of Concurrent Laparoscopic Gastric Band (LAGB) + HHR

##### GORD

Pre- and postoperative GORD rates were recorded in 2 trials. Ardestani et al. saw rates half in both HHR and non-HHR groups (52% vs 53%) [31]. Long et al. saw the GORD rates drop with concurrent HHR; however, with LAGB alone, GORD rates increased [32]. Patients using anti-reflux medication increased from 29.5 to 55.7%. The heterogeneity was significant (*I*^2^ = 90%; *P* = 0.002). The overall effect of concurrent HHR on LAGB led to a 63% reduction in GORD rates (OR = 0.37; 95% CI: 0.35, 0.39).

##### Reoperation

Three trials, involving 34903 patients, reported the rate of reoperation following concurrent HHR+LAGB compared to LAGB alone control. The heterogeneity of the studies was not significant (*I*^2^ = 0%; *P* = 0.48). All 3 studies reported a reduction in the reoperation rate among HHR+LAGB patient compared to patients who had LAGB alone. This meta-analysis showed that the reoperation rate was halved (51%) in the concurrent HHR (OR = 49; 95% CI, 0.37, 0.66).

##### Readmission, Bleeding, and Infection

Readmission, bleeding, and infection were not recorded in the LAGB trials.

##### Mortality

Mortality was recorded in 2 trials. Al-Haddad reported zero mortality in both groups, whilst Ardestani *et al.* reported a mortality rate of 0.02%both LAGB+HHR and LAGB alone groups [31] .

##### Length of Stay

Length of stay (days) was recorded in 2 trials [31, 33]. Length of stay did not significantly differ between the concurrent HHR+LAGB and LAGB alone groups.

## Discussion

To the best of our knowledge, this is the first systematic review to collate the postoperative outcomes of three types of MBS when combined with HHR. The information collected and presented provides a guide to the efficacy and safety of these procedures. We report that combining HHR with MBS appears both safe and feasible for LSG. GORD is the most prominent postoperative issue following LSG. The NBSR data has reported that LSG alone increases GORD rates from 23 to 31%, indicating synchronous HHR should be considered with LSG [9]. In this systematic review, concurrent HHR has been found to improve GORD rates in 9 of 11 studies with an overall reduction of 51% of GORD rates. Likewise, a systematic review performed on the impact of concurrent HHR with LSG on GORD by Chen *et al.* established a positive effect on weight loss, GORD resolution and GORD-HRQL [10]. Reduction in GORD symptoms may be anticipated for several reasons following LSG: reduced gastric volume, quicker emptying, a reduction in acid production and a decreased IAP. However, concurrent HHR help to combat postoperative GORD by re-establishing the anti-reflux mechanism through the restoration of the angle of His and the intra-abdominal position of lower oesophageal sphincter. LSG with HHR however is associated with a slight increased risk of postoperative intervention and readmission rate. Hence, this minimally higher early complication rates are of relatively low morbidity a synchronous approach can be recommended for morbidly obese patients over a staged surgery or conservative management approach [5]. Additionally, the sleeve is not anchored and has the diameter similar to that of the hiatal opening; hence, it is vulnerable to herniation if a HH is left unrepaired, and so serious complications can arise.

LSG has become the most popular choice of bariatric surgery worldwide due to the simplicity of technique, its success in co-morbidity resolution and overall low mortality rate, 0.04% (NBSR). In this study, a marginal increase in mortality risk was observed (OR = 1.23; 95% CI, 0.69, 2.19) when LSG was performed concurrently with HHR. Therefore, with increased research and consolidation of the correct technique for this approach, the rate of mortality should decrease in future.

When performed simultaneously, the short-term outcomes of concurrent RYGB with HHR highlighted favourable results in patients with obesity, in this systematic review. There were no differences in morbidity and mortality as well as other measured outcomes. Similarly, LoS, readmission, and reoperation were all noted in these studies to not be adversely affected.

Society of American Gastrointestinal and Endoscopic Surgeons (SAGES) has established LRYGB as the MBS of choice for the management of GORD in patients with obesity. Frezza *et al.* demonstrated a reduction in heartburn symptoms from 87 to 22% and reduced anti-reflux medication use [12]. De Groot *et al.* also conducted a systematic review into the effect of MBS on GORD; LRYGB was observed to have the most significant impact compared to other MBS [34]. This was demonstrated in another study, where 8 conversions from LSG to LRYGB took place due to recurrent GORD symptoms [24]. Concurrent LRYGB and HHR was seen to narrowly reduce the rates of mortality compared to LRYGB alone (OR = 0.97; 95% CI, 0.27, 3.45). Therefore, this result shows no increased risk of serious adverse complication or risk of the operation when performed with HHR. Reoperation rates were larger compared to LSG but no overall impact from HHR. Shada *et al.* suggested LRYGB with HHR was to be associated with greater morbidity, readmission and reoperation when compared to LSG with HHR [11]. However, the NBSR data showed LRYGB alone has a risk 1.3% reoperation rate compared to LSG’s 0.2%. Therefore, the added HHR is likely not to be responsible.

LAGB meanwhile was demonstrated to be both safe and feasible in all 4 studies. Reoperation was observed to be much lower in all 3 studies when compared to their LAGB controls. Concurrent HHR had a 51% reduced rate of reoperation compared to LAGB alone (OR = 0.49; 95% CI, 0.37, 0.66). Long *et al.* demonstrated HHR have a 3.5% rate of PD versus 10.5% in LAGB alone [32], whilst Fielding *et al.* demonstrated lower reoperation rates of 1.7% with HHR compared to 5.6% in the LAGB control [33]. These results suggest HH plays a role in the recurrence or occurrence of PD and gastric prolapse. The weight-loss post-LAGB diminishes the epiphrenic fat pad, exposing and enlarging the HH, in turn causing PD or slippage. Crural reinforcement results in a significant decrease. The most recent recordings of the incidence of LAGB being performed per year shows LAGB to be the least performed bariatric surgery out of the 3 included in this review (NBSR 2020). Rates have largely decreased due to high rates of reoperation. However, with the introduction of additional HHR, reoperation rates decreased and therefore may allow this procedure to be more widely performed.

When LAGB was performed concurrently with HHR, GORD rates were seen to drop by 63% (OR = 0.37; 95% CI, 0.35, 0.39). The NBSR data states LAGB alone to reduce GORD rates by 8%; therefore, improved results are experienced. This may be due to an overall restoration in anatomy, which can in turn prevent pouch dilatation and so gastric prolapse. The LOS disruption experienced in PD and the postulated high-pressure system created in LAGB can in turn precipitate GORD [35].

HH size may affect a surgeon’s decision on whether to perform HHR at the time of MBS. Small HH can be considered as clinically “silent” and therefore repair unnecessary. However, a consensus from these studies is that due to the weight loss experienced post-MBS, the HH can be observed to enlarge due to fat loss around the GOJ, and so lead to the intra-thoracic migration of the stomach or other organs. Therefore, further research and a quantitative scale for size should be implemented into future studies. At this time and from the information provided HH of any size should be repaired at the time of MBS to prevent further complication.

### Further Research

This study was unable to produce solid evidence to fully support the implementation of HHR with MBS due to the available data being too inconsistent to allow for a full meta-analysis of all outcomes. A new, large population-based cohort study, accounting for different repair techniques, with objective measurement of symptoms (including 24-h pH monitoring for GORD status), needs to be performed. Within this study, a quantitative HH size scale should be administered to differentiate variation in safety and clinical outcomes of different-sized HH. The data should allow for a meta-analytical review of all measured outcomes. This review should also include outcome measures such as PD and baseline co-morbidities, allowing other health measurements such as diabetes remission to be investigated.

## Conclusion

To conclude, this systematic review features data collected from 17 studies and determines that HHR may be performed safely and effectively at the time of MBS. Considering the findings, we recommend that surgeons consider concurrent HHR and MBS. Individual patient suitability and surgeon experience are important considerations for each case. It is important to discuss the potential risks and carefully follow postoperative instructions to ensure the best possible outcome. Although there is a large volume of published data regarding this topic, further research is needed to standardise measurements and gain further insight into the postoperative outcomes.

### Supplementary Information

ESM 1 (DOCX 345 kb)
