# Supplementary material for: Outcomes of Concurrent Hiatus Hernia Repair with Different Bariatric Surgery Procedures: a Systematic Review and Meta-analysis
Source: Obes Surg. 2023 Nov 2;33(12):3755–66. doi: 10.1007/s11695-023-06914-7 (PMC10687114; doi:10.1007/s11695-023-06914-7)
Supplement: Supplementary file 1 — (DOCX 345 kb) [file 11695_2023_6914_MOESM1_ESM.docx]

Supplement 1:

PubMed Search Strategy:

((("Hernia, Hiatal"[Mesh]) OR (hiatus hernia repair)) OR (Paraesophageal Hiatal Hernia)) AND (("Bariatric Surgery"[Mesh]) OR metabolic surgery OR weight loss surgery)

("hernia, hiatal"[MeSH Terms] OR ("hernia"[All Fields] AND "hiatal"[All Fields]) OR "hiatal hernia"[All Fields] OR ("hernia"[All Fields] AND "hiatal"[All Fields]) OR "hernia hiatal"[All Fields] OR (("hernia, hiatal"[MeSH Terms] OR ("hernia"[All Fields] AND "hiatal"[All Fields]) OR "hiatal hernia"[All Fields] OR ("hiatus"[All Fields] AND "hernia"[All Fields]) OR "hiatus hernia"[All Fields]) AND ("repairability"[All Fields] OR "repairable"[All Fields] OR "repaire"[All Fields] OR "repaired"[All Fields] OR "repairment"[All Fields] OR "wound healing"[MeSH Terms] OR ("wound"[All Fields] AND "healing"[All Fields]) OR "wound healing"[All Fields] OR "repair"[All Fields] OR "repairing"[All Fields] OR "repairs"[All Fields])) OR ("hernia, hiatal"[MeSH Terms] OR ("hernia"[All Fields] AND "hiatal"[All Fields]) OR "hiatal hernia"[All Fields] OR ("paraesophageal"[All Fields] AND "hiatal"[All Fields] AND "hernia"[All Fields]) OR "paraesophageal hiatal hernia"[All Fields])) AND ("bariatric surgery"[MeSH Terms] OR ("bariatric"[All Fields] AND "surgery"[All Fields]) OR "bariatric surgery"[All Fields] OR ("bariatric surgery"[MeSH Terms] OR ("bariatric"[All Fields] AND "surgery"[All Fields]) OR "bariatric surgery"[All Fields] OR ("metabolic"[All Fields] AND "surgery"[All Fields]) OR "metabolic surgery"[All Fields]) OR ("bariatric surgery"[MeSH Terms] OR ("bariatric"[All Fields] AND "surgery"[All Fields]) OR "bariatric surgery"[All Fields] OR ("weight"[All Fields] AND "loss"[All Fields] AND "surgery"[All Fields]) OR "weight loss surgery"[All Fields]))

Supplement 2:


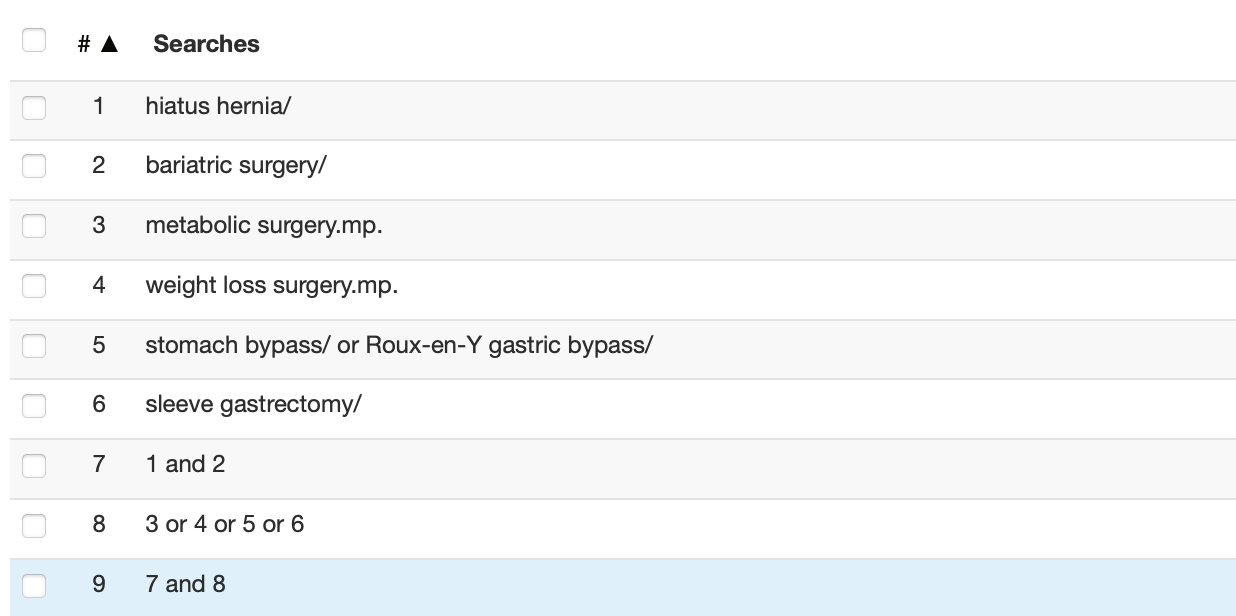
Medline

Supplement 3:

Embase


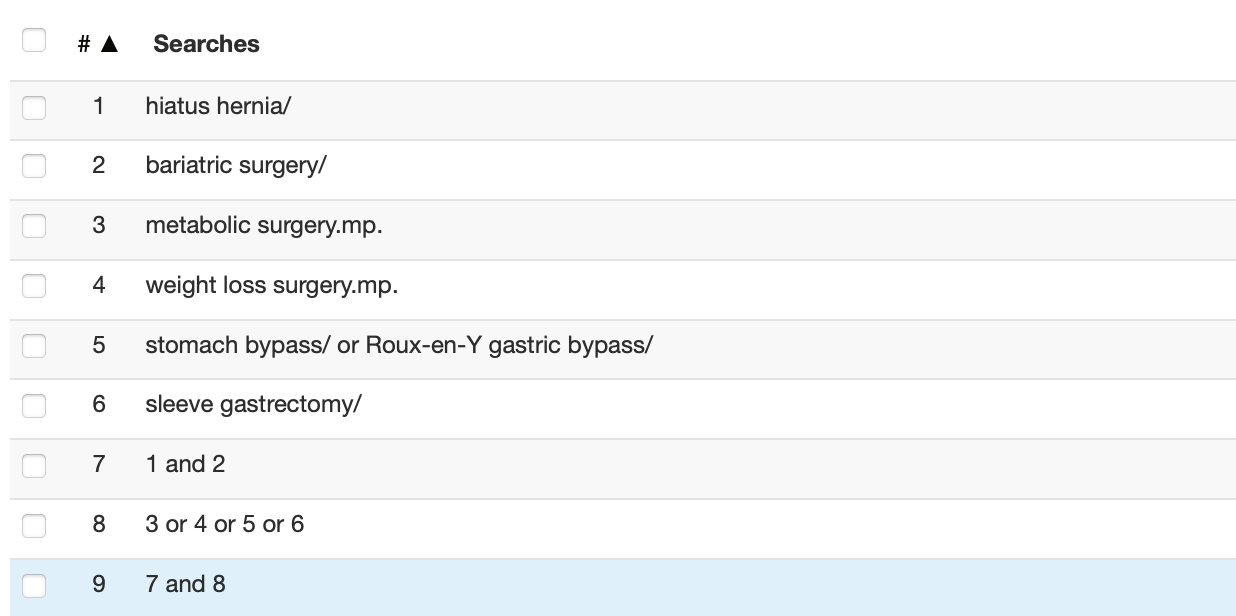


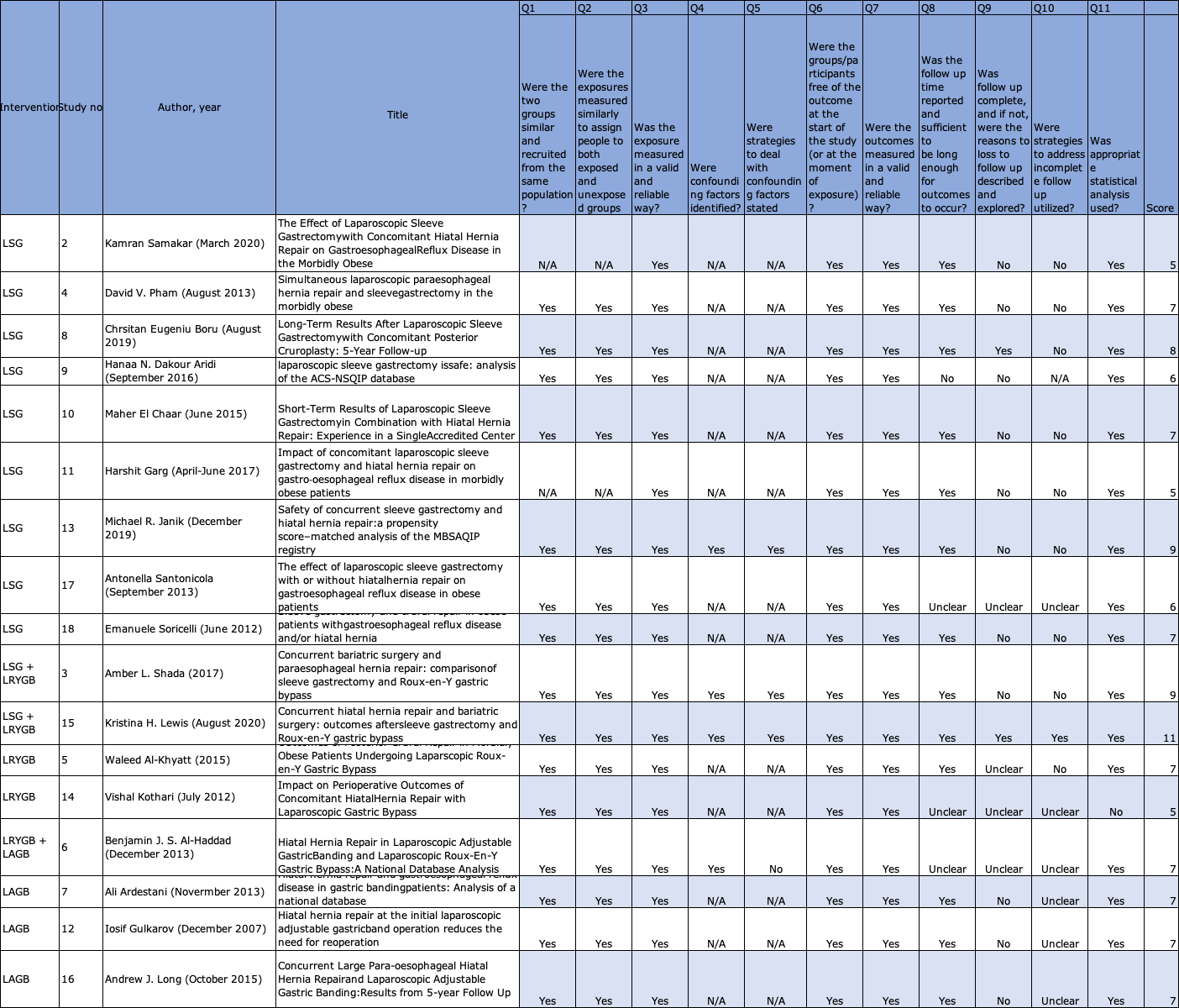
Supplement 4:


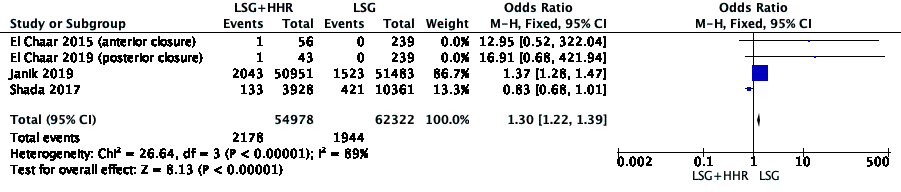
Supplement 5:
